# Full transcriptome analysis of Chinese Hamster Ovary cell lines producing a dynamic range of Coagulation Factor VIII

**DOI:** 10.1186/1753-6561-7-S6-P56

**Published:** 2013-12-04

**Authors:** Christian S Kaas, Claus Kristensen, Jens J Hansen, Gert Bolt, Mikael R Andersen

**Affiliations:** 1 Department of Mammalian cell technology, Novo Nordisk A/S, Maaloev, 2760, Denmark; 2 Center for Microbial Biotechnology, Technical University of Denmark, Kgs Lyngby, 2800, Denmark

## Background and novelty

Coagulation Factor VIII (FVIII) is an essential cofactor in the blood coagulation cascade. Inability to produce functional FVIII results in haemophilia A which can be treated with recombinant FVIII [1]. Chinese Hamster Ovary (CHO) cells are the most used cell line for producing complex biopharmaceuticals due to its ability to perform complex post-translational modifications. When mammalian cells overexpress a protein like FVIII they will adapt by regulating various proteins and pathways to support synthesis/production of this protein. Yields of FVIII produced in CHO are low and for this reason a greater understanding of what constitute a high producing cell line is desired. In this study a full transcriptome analysis was undertaken in order to analyze the differences between high and low producers of FVIII

## Experimental approach

The FVIII gene was introduced into CHO-DUKX-B11 cells and a stable pool was generated by selection with MTX. A number of subclones were analysed and 3 high producing clones, 3 medium producers and 3 low (~0) producer clones were isolated. These 9 clones were grown in shake flasks in batch culture. During the cultivation essential metabolites were monitored as well as cell number and viability. RNA was extracted after 48 hours of cultivation and sequenced using the Illumina HiSeq system. Reads were processed and aligned to the CHO-K1 genome [2] using Tophat2 and expression levels were deduced using htseq

## Results and discussion

**Figure 1:**
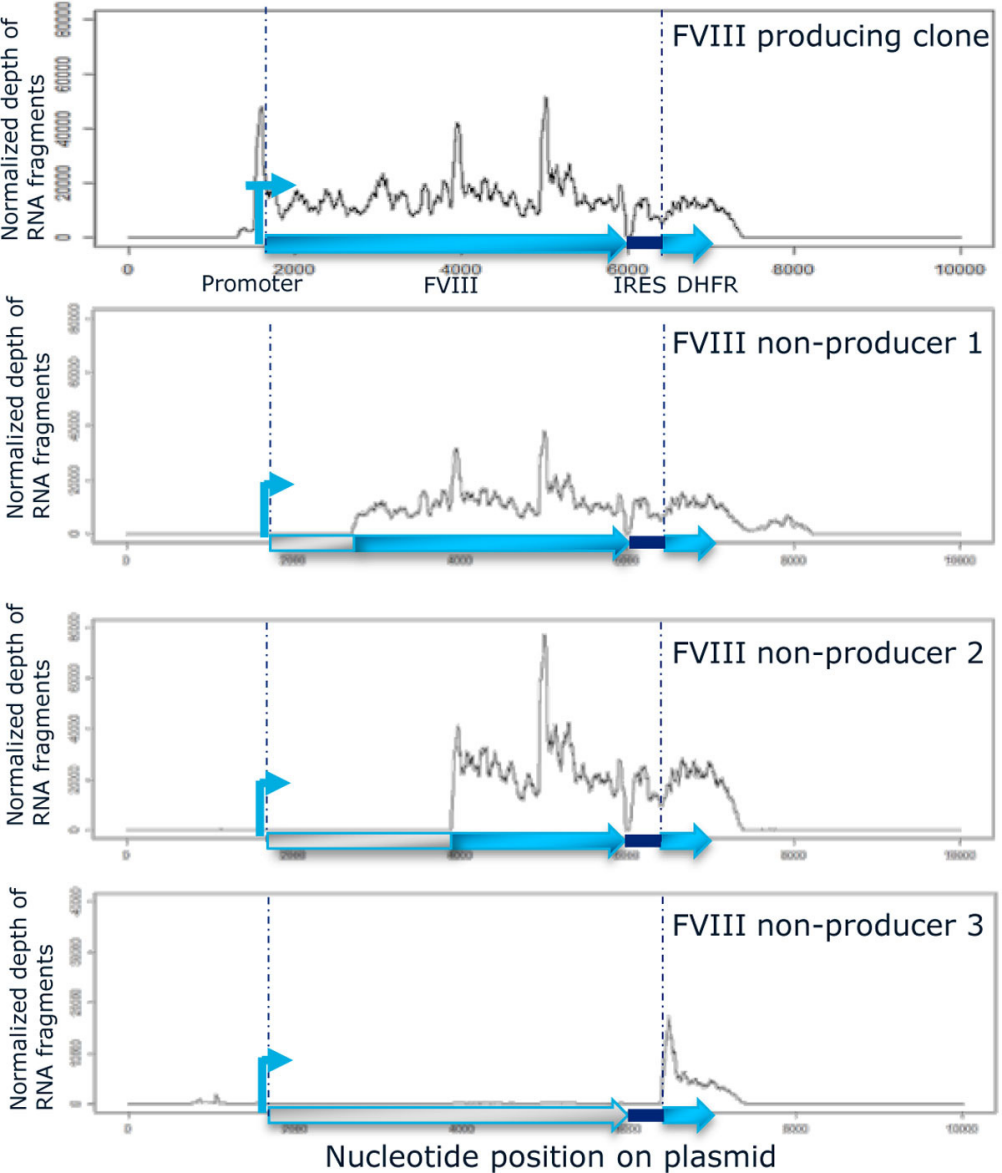
**Depth of sequenced reads at every position of the FVIII gene**. It is seen that the 3 non-producing clones transcribe 5'-truncated RNA species. This would explain the phenotype of no FVIII protein production but growth under MTX selection as the IRES element containing DHFR is still transcribed.

Experiments showed that 48 hours into the cultivation cells were seen to grow in the exponential phase in media still containing sufficiently high amounts of glutamine and low amounts of lactate. Furthermore, a significant difference in FVIII levels was detected at this time in the media of cells from the different groups and for this reason this time point was chosen for extraction of RNA. 1677 genes were found to be differentially expressed in high vs non-producing clones. Among these, genes involved in oxidative stress were seen to be enriched (p = 1.74 × 10^-6^). This finding is strengthened by the work by Malhotra *et al *[3] showing that CHO cell lines activate the oxidative stress response when producing FVIII, which might induce apoptosis. The non-FVIII-producing clones were seen to express predominantly truncated FVIII-DHFR mRNAs (Figure 1) explaining the phenotype for growth in media containing MTX selection but no functional FVIII expressed. Further analyses are ongoing.
